# Direct detection and quantification of *Toxoplasma gondii* in meat samples from feral raccoons (*Procyon lotor*) in Germany by magnetic-capture real-time PCR

**DOI:** 10.1007/s00436-022-07730-1

**Published:** 2022-11-19

**Authors:** Lydia Engel, Ahmad Hamedy, Martin Koethe

**Affiliations:** Institute of Food Hygiene, Faculty of Veterinary Medicine, Leipzig University, An den Tierkliniken 1, 04103 Leipzig, Germany

**Keywords:** Wildlife, Game, Zoonosis, DNA, MC-PCR

## Abstract

Because the number of wild raccoons in Germany is increasing constantly, it appears to be economic reasonable to use their meat as food. For this purpose, it is essential to generate data regarding the pathogen load of the meat to be consumed and handled. It is known that raccoons, particularly in Germany, show a high seroprevalence of *Toxoplasma gondii*. Because serological data only indicates contact of a host to a parasite additional direct detection is needed to prove presence of parasitic stages in particular tissues. Therefore, a total of 150 samples from raccoons with known serostatus were tested and quantified using magnetic-capture real-time PCR for *Toxoplasma gondii.* As it represents potentially consumption-relevant parts of raccoons, meat from forelimb and hindlimb was examined. Samples were stratified into three groups based on the animals’ serostatus (each 50 negative, low positive, and high positive). All samples from seronegative animals were found negative by MC-PCR as well. In a total of 56 meat samples from 100 seropositive animals, *T. gondii* DNA was detected. Statistically significant more samples were positive by MC-PCR in the high positive than in the low positive serostatus group (38/50 vs. 18/50, *p* < 0.0001). Furthermore, samples from the former group were also found to have statistically significant higher DNA equivalent values compared to samples from the low positive serostatus group (*p* < 0.0001). These results suggest that meat from seropositive raccoons may contain considerable numbers of *T. gondii* presenting a potential public health risk for humans whilst handling and consumption.

## Introduction

*Toxoplasma gondii* is a coccidian parasite, which can probably infect all warm-blooded animals as intermediate hosts and cats as definitive hosts (Tenter et al. 2000). Whilst only Felidae excrete oocysts, all warm-blooded animals can accommodate tissue cysts in brain or muscles (Dubey 2022). Humans can become infected by a wide range of exposures. Tissue cysts containing infectious bradyzoites can be taken up through contaminated raw or undercooked meat of food-producing animals or wildlife (Jones and Dubey 2012). Additionally, the infectious oocysts can be transmitted through ingestion of contaminated food, soil, or water by cat faeces (Jones et al. 2014). Toxoplasmosis usually stays subclinical or is accompanied by mild symptoms of illness like fever, malaise, and lymphadenopathy. However, in people with pre-existing disease or immunosuppression, severe disease processes with neurologic involvement, most commonly due to encephalitis, and up to mortality may occur (Jones et al. 2014). Primary maternal infection can lead to transmission of the parasite to the foetus and a wide range of clinical manifestations like ocular disease, hydrocephalus, and intracerebral calcifications, or even abortion may arise (Dubey 2022). Due to its medical and veterinary importance, *T. gondii* is considered one of the most well-studied parasites (Dubey 2022). However, many studies on food-producing animals and game are mainly focusing on the presence of antibodies (Berger-Schoch et al. 2011; Bier et al. 2020; Lundén et al. 2002; Račka et al. 2015). Furthermore, economic and public health aspects regarding wild canids and other carnivores like raccoons, which are mainly raised for fur or meat production, have also been discussed recently (Dubey et al. 2021).

The raccoon (*Procyon lotor*) originated in North America and became established in Europe at the beginning of the past century (Kauhala 1996; Lutz 1995). As the spread increased strongly from the 1970s onwards (Lutz 1995), population management measures have become more and more important since this uncontrolled distribution (Beltrán-Beck et al. 2012; Salgado 2018). Along to this, the German Hunting Association (DJV) has recorded a strong increase in the annual hunting bag data, from about 8000 in the year 1999/2000 to more than 200,000 in 2019/2020 (German Hunting Association 2021). With the increasing popularity of eating game (German Hunting Association 2019), the use of raccoon meat may also be increasingly conceivable, as well as in other countries such as the USA, even though raccoon is still consumed less than the usual game species there (Burger 2000; Gaines et al. 2000; German Hunting Association 2020; Goguen and Riley 2020). However, raccoon meat consumption is rather rare in Germany, but is considered to be a delicacy amongst hunters (German Hunting Association 2020).

Using serological analyses, a large number of individuals can be examined at low cost, so these are preferred for screening (Opsteegh et al. 2011). A positive serological result only indicates exposure to the parasite, but does not confirm presence of the parasite in host tissues (Dubey 2022). However, the presence of antibodies may indicate an infection risk by consuming meat, if there is a strong correlations between the presence of antibodies against *T. gondii* and tissue cysts (Opsteegh et al. 2011). This correlation can be different in the various host species. Whilst strong correlations were found for pigs and sheep (Dubey et al. 2008; Gamble et al. 2005; Opsteegh et al. 2010), this was not the case for cattle (Opsteegh et al. 2011). Therefore, it is needed to examine such correlation for a specific species, i.e. the raccoon. Additionally, different parts of the organism may be affected to varying degrees by tissue cysts (Dubey 2022; Koethe et al. 2015; Tenter et al. 2000). Thus, to assess the human health risk, *T. gondii* infestation of consumption-relevant meat parts from raccoons needs to be evaluated.

Recent studies demonstrated a high seroprevalence of *T. gondii* in German raccoons (Engel et al. 2022; Heddergott et al. 2017; Heddergott and Müller 2020). However, seropositive animals do not immediately pose a risk to humans, as they do not always harbour tissue cysts with infectious parasites (Halos et al. 2010). Nevertheless, when performed conventionally, essential direct detection is laborious, costly, and insufficiently sensitive (Algaba et al. 2017). Due to this, there is not much published information on PCR for *T. gondii* in rarely consumed species like raccoons. For naturally infected feral raccoons, one study was conducted in Poland using a conventional PCR to analyse brain, lung, and heart of the animals for *T. gondii* DNA (Kornacka et al. 2018). However, conventional DNA extraction methods are not very sensitive as only small amounts of tissue with a maximum of 50 mg can typically be examined and the distribution of *T. gondii* tissue cysts is non-homogeneous in hosts (Opsteegh et al. 2010). Due to a lack of sensitivity, Opsteegh et al. (2010) developed a method in which up to 100 g of tissue can be analysed for the presence of *T. gondii* DNA, by sequence-specific DNA extraction using magnetic capture followed by real-time PCR. Since then, this magnetic-capture PCR (MC-PCR) has been successfully applied to various animal species or animal products with several minor modifications and different experimental setups (Gomez-Samblas et al. 2015; Hosein et al. 2016; Juránková et al. 2013; Koethe et al. 2015; Nicholas et al. 2018; Stollberg et al. 2021). Additionally, real-time quantitative PCR (qPCR) offers the advantage for quantification of parasite DNA. In general, PCR methods itself are sensitive, as they can detect DNA from as few as one tachyzoite, specific, and enable quick diagnosis (Dubey 2022). Since there are different types of PCR, quantitative MC-PCR was found to be the most sensitive method for detecting *T. gondii* DNA (Stollberg et al. 2021). Because of the high seroprevalence of German raccoons (Engel et al. 2022; Heddergott et al. 2017; Heddergott and Müller 2020), this study focuses on direct detection of *T. gondii* DNA in consumption-relevant raccoon meat by MC-PCR to provide more relevant information for future human health risk evaluation. To the authors’ knowledge, this is the first time MC-PCR was performed on raccoon meat.

## Material and methods

### Meat samples

Raccoons were gathered for sampling as previously detailed by Engel et al. (2022). Based on serological investigations performed previously by Engel et al. (2022), according to enzyme-linked immunosorbent assay (ELISA) results, animals were stratified into three groups. One group comprised seronegative animals with a sample to positive control (*S*/*P*) ratio < 8%. The other groups comprised either low positive animals with an *S*/*P* ratio between 30 and 60% or high positive ones with an *S*/*P* ratio > 120%. From each group, 50 raccoons were randomly chosen and sampled for molecular diagnostics. For sampling, each one previously frozen forelimb and hindlimb of every animal were defrosted for 2 days in a refrigeration chamber at 1 °C.

### Magnetic-capture DNA extraction and real-time PCR

Samples were prepared and DNA was extracted by magnetic capture as described by Opsteegh et al. (2010) using minor modifications (Koethe et al. 2015) as well as some additional slight deviations. Approximately 100 g (67.1–116.2 g) meat sample (free of fat and connective tissue), which was composed of approximately 75% of the hindlimb and 25% of the forelimb due to meat availability, was used for further examination.

*T. gondii* tachyzoites (ME 49) were kindly provided from Institute of Parasitology, Faculty of Veterinary Medicine, Leipzig University, in Dulbecco Modified Eagle Medium (including 10% foetal bovine serum, 1% penicillin/streptomycin). To generate quantification standards and control samples for each magnetic-capture (MC) DNA extraction, meat from nine seronegative raccoons (*S*/*P* ratio < 4%) was demonstrated *T. gondii* DNA-free by MC-PCR, pooled, and split into 10-g aliquots. Tachyzoites were serially diluted in phosphate buffered saline (PBS) and added at a final concentration of 10^6^ to 10^2^ per 10 g of negative raccoon meat aliquot for quantification standards. For these standards, DNA was extracted by magnetic capture and thereupon included in every PCR run to calculate the amount of *T. gondii* in the examined sample. Additionally, for every MC-DNA extraction approach a 10-g meat aliquot was spiked with 10^3^ tachyzoites to serve as positive extraction control. Another 10-g meat aliquot without tachyzoites was included as negative extraction control. By comparing the resulting sample cycle threshold value (*C*_t_ value) with the standards the quantity of *T. gondii* genome equivalents of each sample was calculated (StepOnePlus software, Life Technologies, Germany) and adjusted to determine the number of genome equivalents per 100 g of meat, expressed as log_10_ values. Qualitatively, MC-PCR results with *C*_t_ values < 35 were considered positive, and samples with *C*_t_ values > 40 were regarded as negative. From samples with *C*_t_ values between 35 and 40, the respective amplification curves were visually inspected and considered negative when no typical amplification course was observed.

### Statistics

The statistical analyses were performed by Prism9 Software (GraphPad Software, LLC, USA). Low and high positive serostatus groups were compared by chi-square test regarding qualitative PCR results. To analyse for differences between these two groups in respect to genome equivalents per 100 g meat, quantitative PCR results were compared by *t*-test after confirmation of Gaussian data distribution. The Spearman correlation coefficient (*r*_S_) was determined to describe the relationship between *S*/*P* ratio and DNA equivalents present in 100 g meat. In general, *p* < 0.05 was regarded statistically significant.

## Results

In 56 of 150 examined raccoon meat samples *T. gondii* DNA was detected by MC-PCR. All of the 50 serologically negative animals were also found to be negative for *T. gondii* DNA. Out of the serologically low positives (30–60% *S*/*P* ratio), 18 samples (36%) were MC-PCR positive, whilst 38 out of the 50 analysed samples from the high positive serostatus group (76%) were found to contain *T. gondii* DNA. Statistically, significantly more positive samples were detected in the high positive serostatus group (chi-square test, *p* < 0.0001). Detailed results are shown in Table 1.

**Table 1:** Qualitative MC-PCR results of raccoon meat samples grouped by serostatus of the originating animals

MC-PCR result	Serological result (*S*/*P* ratio)			
	Negative (< 8%)	Low positive (30–60%)	High positive (> 120%)	Total
Positive	0	18	38	56
Negative	50	32	12	94
Total	50	50	50	150

**Fig. 1:**
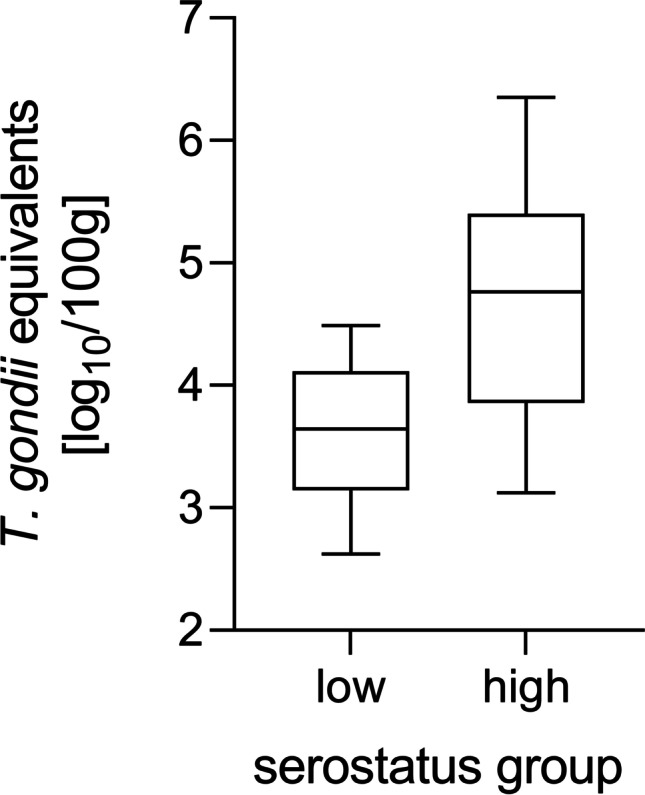
Box-and-whisker diagram of quantitative MC-PCR result distribution in the low and high positive serostatus groups. Minimum, 25% percentile, median, 75% percentile, and maximum are displayed from 18 (low positive serostatus) or 38 (high positive serostatus) MC-PCR positive samples

In samples with high *S*/*P* ratios statistically significant higher DNA equivalent values were detected compared to MC-PCR positive samples with lower S/P ratio (4.688 vs. 3.610 log_10_; *p* < 0.0001). In general, the amount of *T. gondii* DNA equivalents amongst the MC-PCR positive samples ranged from 2.622 log_10_ (*S*/*P* ratio of this sample: 30.02%) to 6.352 log_10_ (*S*/*P* ratio: 177%) per 100 g meat. Details on quantitative results are shown in Fig. 1.

**Fig. 2:**
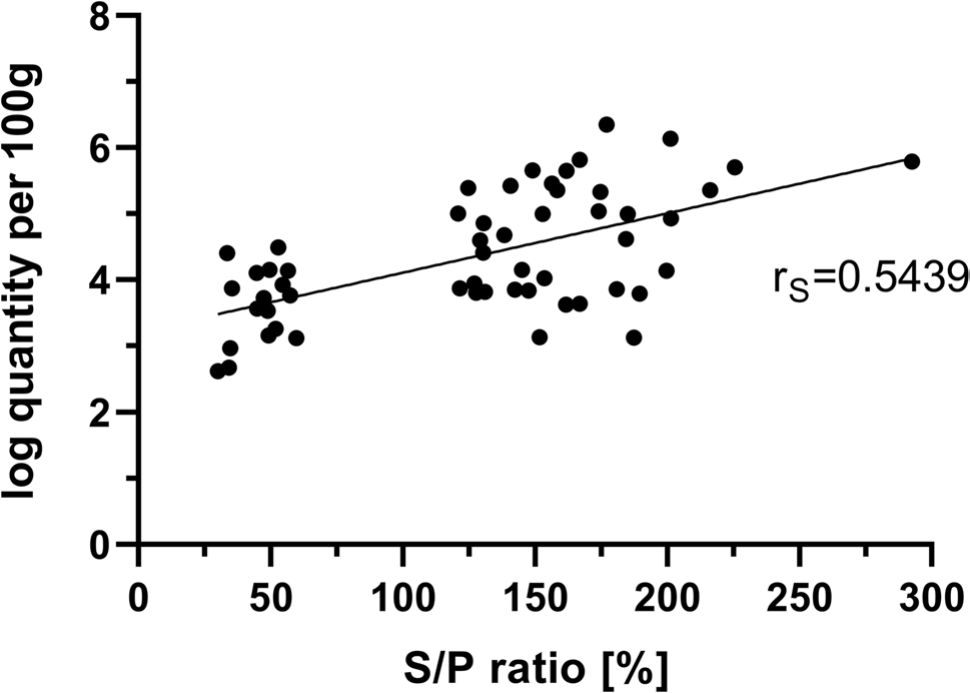
Scatter diagram of quantitative MC-PCR results (log_10_ values) depending on serological results (*S*/*P* ratio). Best-fit line from linear regression and Spearman correlation coefficient (*r*_S_) are displayed

Based on the quantitative MC-PCR results of the herein analysed samples, there was a positive correlation between *S*/*P* ratio and DNA equivalents (*r*_S_ = 0.5439, *p* < 0.0001), which is illustrated in Fig. 2.

## Discussion

Generally, in many studies *T. gondii* DNA was detected in samples from seropositive animals, including wildlife (Dubey 2022). Particularly for Germany, foxes (*Vulpes vulpes*) (Herrmann et al. 2012),wild boars (S*us scrofa*), roe deer (*Capreolus capreolus*), and red deer (*Cervus elaphus*) were shown to contain *T. gondii* DNA (Stollberg et al. 2021). For raccoons, Kornacka et al. (2018) recently detected *T. gondii* DNA in typical predilection sites of seropositive animals by conventional PCR, especially in the brain but also in lungs and hearts. Based on only a few samples, they reported detection of *T. gondii* DNA in 50% (3/6) of serologically positive animals originating from Poland and Germany. Using the more sensitive MC-PCR method, we detected *T. gondii* DNA in meat samples of 56% (56/100) of serological positive raccoons. Further investigations in German wildlife made comparable findings, where approximately 52% (24/46) of wild boar and 41% (7/17) of roe deer samples were serological positive as well as DNA positive by PCR (Stollberg et al. 2021).

A higher agreement was detected in Canadian foxes with approximately 69% (11/16) of serological positives which were positive by MC-PCR as well (Nicholas et al. 2018). A much lower proportion of DNA positives in serological positive red foxes from Italy (approximately 8%, 8/102) was reported by Verin et al. (2013). Similar low agreements were found for German foxes by Herrmann et al. (2012), who observed DNA in 48 out of 301 (16%) serological positive animals by conventional PCR. Analysing German red deer, Stollberg et al. (2021) also found a comparable low proportion of PCR positive results on seropositive animals (25%). However, only single animals of red deer have been tested (*n* = 4) in that study. A high detection rate of DNA in serologically positive samples, e.g. as detected by Nicholas et al. (2018) with 69%, might be caused by different factors. They also used the sensitive MC-PCR method for DNA detection but examined tissues like brain and heart that are known to be predilection sites in many species (Juránková et al. 2014a, 2015; Koethe et al. 2015; Santoro et al. 2019), which might account for the higher detection rate compared to our results in skeletal muscle samples. MC-PCR is still not commonly used in animals or meat products, as it is a very laborious and expensive investigation. However, this type of DNA extraction was repeatedly found to be considerably more sensitive than conventional ones (Juránková et al. 2014b; Opsteegh et al. 2010). Using conventional PCR can, therefore, be a reason for lower DNA detection rates in seropositive animals.

To detect *T. gondii* DNA in meat products, especially for products that are usually uncooked, it is possible to apply MC-PCR as well. For example, it was successfully deployed for the examination of serrano ham, which was found to contain *T. gondii* DNA with a prevalence of approximately 9% (Gomez-Samblas et al. 2015). However, MC-PCR is performed mostly on predilection sites of *T. gondii*, which are expected to have a higher prevalence than examining skeletal muscle. Whilst this is reasonable for general parasitic investigations the aim of this study was to contribute knowledge on prevalence and quantity of *T. gondii* in raccoon meat parts that are relevant for potential consumption. Different sample material may also account for differences in DNA detection rates.

Additionally, differences on the level of agreement between serostatus and DNA prevalence may be due to the corresponding antibody level and the applied method of serological examination. In this study, animals were stratified according to serological status into serological low positive and high positive based on previous ELISA results (Engel et al. 2022). Different serological methods may include distinct discrimination levels, which could lead to a different categorization of serological results and, thus, to a different level of agreement. To date, the dynamics of antibody response following contact with the parasite in raccoons are not well known. Thus, the extent to which a current serostatus information is reliable for the infection status of a raccoon is uncertain.

All serologically negative raccoons examined in this study also proved negative in MC-PCR. For raccoons, Kornacka et al. (2018) identified 44% (15/34) serologically negative animals to contain *T. gondii* DNA in animal predilection sites, which is contrary to our observations, but is similar to previous investigations for other animals. Nicholas et al. (2018) found 30% (7/23) of serological negative foxes in Canada to be positive by MC-PCR for *T. gondii* DNA. This might be because they examined the predilection sites of animals, where tissue cysts are usually most common. Furthermore, we only investigated animals with a very low *S*/*P* ratio (< 8%) for the seronegative group, whilst all animals with an *S*/*P* ratio < 25% are regarded negative according to the manufacturers’ instructions. Kornacka et al. (2018) used the same test and it must be assumed that seronegative animals from their study have a broader *S*/*P* range compared to the seronegative animals in our study. Similar findings were made previously for other animals, using serological examinations and bioassay. Seronegative pigs rarely also harboured infectious *T. gondii* stages (Dubey et al. 1995) which was later discussed to be because of a very recent infection or of a decrease of antibodies to an undetectable level (Dubey 2022).

In this study, we detected a positive correlation between antibody level and presence of *T. gondii* DNA. For 36% of the serologically low positive animals and for 76% of the serologically high positive animals, *T. gondii* DNA could be detected. Similar observations were made for other species as well. In chickens, a correlation was observed between serological status and pathogen isolation. The frequency of *T. gondii* isolation increased sharply with rising MAT titres; whilst only 61% were isolated at low titres, it was already up to 75% at high titres using a bioassay (Dubey et al. 2016). This leads to the conclusion that higher seroprevalence titres are related to higher parasite loads (Dubey 2022).

However, results strongly depend on the examined tissue, DNA extraction method, PCR method (Dubey 2022), and the amount examined, as it is known that *T. gondii* is not homogeneously distributed in organisms (Opsteegh et al. 2010). For detection of infectious parasites, a bioassay, e.g. together with a cell culture, is necessary which is very time-consuming, associated with high costs, and requires the use of laboratory animals (Algaba et al. 2017; Dubey 2022). Using PCR, only DNA detections are obtained. The material costs of MC-PCR are higher than for conventional DNA extraction, which is usually performed with a special kit but they are not as high as for a bioassay (Opsteegh et al. 2010). Thus, MC-PCR can be used as an alternative to bioassay in respect to detection and genotyping of *T. gondii*, and to quantify the organism in meat samples of various source (Opsteegh et al. 2010).

For wild animals in Germany, the examination of tissue by MC-PCR showed the best agreement for DNA detection in relation to serological examinations (Stollberg et al. 2021). For sheep and lambs, there was a statistically significant increase in the probability of isolating *T. gondii* in skeletal muscle with increasing sample size per animal (Rani et al. 2020). Since in conventional PCR only a few milligrams or up to a few grams of meat can be examined, an MC-PCR has a significantly higher probability for detecting parasite DNA when using up to 100 g meat.

Finally, we observed skeletal muscle tissue as meat for potential human consumption. Since for different animal species brain and heart were found to be the main predilection sites for *T. gondii* tissue cysts (Juránková et al. 2014a, 2015; Koethe et al. 2015; Santoro et al. 2019) we may underestimate the general prevalence of *T. gondii* DNA in raccoons. However, the focus of this study rather was the detection of parasite burden in consumption relevant parts of raccoons to provide knowledge for further public health evaluations on this kind of meat. We found that meat from seropositive raccoons may contain up to about 6 log_10_ of *T. gondii* equivalents. Based on the modelling of Guo et al. (2016) this number would be sufficient for a high probability of infection in humans.

## Conclusion

Previous seroepidemiological research proved a high presence of *T. gondii* in German raccoons. In addition, we showed a high detection rate of *T. gondii* DNA in meat from seropositive raccoons. Seropositive animals could harbour considerable numbers of *T. gondii*, where antibody titre is positively correlated with DNA amount. If the parasites in meat are also likely to be infective, appropriate care must be taken to avoid infection and the meat has to be sufficiently heated before consumption. To better assess the public health risk posed by raccoon meat, further investigations, such as bioassay for evaluation of parasite infectivity, need to be performed.

## Data Availability

The data related to the manuscript will be available upon request to the corresponding author.
